# Severe reflux esophagitis after total gastrectomy successfully treated by transposition of the jejunojejunal anastomosis: a report of two cases

**DOI:** 10.1186/s40792-021-01350-0

**Published:** 2021-12-20

**Authors:** Noriyuki Nishiwaki, Shinji Hato, Tetsuya Kagawa, Tomokazu Kakishita, Isao Nozaki

**Affiliations:** 1 Department of Surgery, National Hospital Organization, Shikoku Cancer Center, 160, Ko, Minamiumemoto-machi, Matsuyama-shi, Ehime-Ken 791-0280 Japan; 2 Department of Gastroenterological Surgery, Fukuyama City Hospital, 5-23-1, Zao-cho, Fukuyama City, Hiroshima Prefecture 721-8511 Japan; 3 Department of Surgery, National Hospital Organization Okayama Medical Center, 1711-1 Tamasu, Kita-ku, Okayama City, Okayama 701-1192 Japan

**Keywords:** Total gastrectomy, Reflux esophagitis, Jejunojejunostomy

## Abstract

**Background:**

Reflux esophagitis after total gastrectomy is often difficult to treat. In this report, we describe two cases of reflux esophagitis that were refractory to medical therapy and successfully treated by transposition of the jejunojejunal anastomosis.

**Case presentation:**

Case 1: A 66-year-old man underwent total gastrectomy and cholecystectomy for gastric cancer, and Roux-en-Y (RY) reconstruction was performed. The pathological diagnosis was T4aN3aM0 stage IIIC. Five months later, esophagogastroduodenoscopy identified reflux esophagitis. Although he was treated with various oral medications and was hospitalized six times, he lost 19 kg of weight. Finally, the patient was reoperated 3 years postoperatively. Intraoperative findings showed that there was no evidence of recurrence or severe adhesions that could have caused obstruction, and the anastomotic distance between the esophagojejunostomy and the jejunojejunostomy was approximately 40 cm. The jejunojejunostomy was re-anastomosed to increase the distance to 100 cm. Two years and 6 months after the reoperation, there was no recurrence of reflux esophagitis, and the patient’s weight increased by 14 kg.

Case 2: A 68-year-old woman underwent total gastrectomy and cholecystectomy for gastric cancer, and RY reconstruction was performed. The pathological diagnosis was T4aN0M0 stage IIB. Similar to Case 1, the patient was diagnosed with reflux esophagitis 5 months later. She lost 23 kg of weight and was reoperated at 6 months postoperatively. Intraoperative findings showed that there was no evidence of recurrence or severe adhesions, and transposition of the jejunojejunostomy was performed to increase the distance between anastomoses from 40 to 100 cm. Two years and 8 months after the reoperation, there was no recurrence of reflux esophagitis, and her weight increased by 15 kg.

**Conclusions:**

Transposition of the jejunojejunostomy was an effective treatment for medication-resistant severe reflux esophagitis after total gastrectomy.

## Background

Reflux esophagitis is a complication that can occur after total gastrectomy [1]. Although Roux-en-Y (RY) reconstruction has significantly decreased the incidence of this complication, when it does occur, it is often difficult to treat [2–4]. In this report, we describe two cases of reflux esophagitis that were refractory to medical therapy and successfully treated by transposition of the jejunojejunal anastomosis.

## Case presentation

**Fig. 1:**
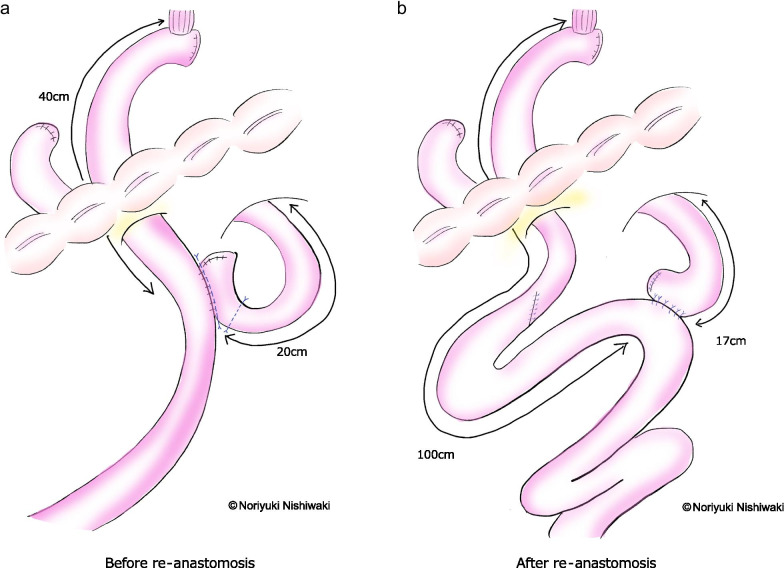
Schemes of intraoperative findings. **a** The anastomotic distance between the esophagojejunostomy and the jejunojejunostomy was about 40 cm. The cutting lines of the jejunum were shown in blue lines. **b** After the jejunojejunostomy was dissected and closed, it was re-anastomosed to make anastomotic distance 100 cm

**Fig. 2:**
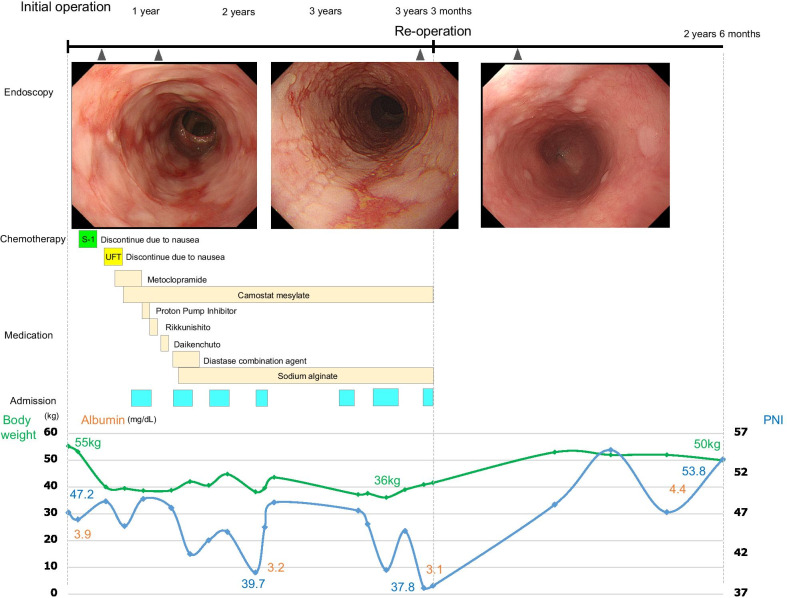
Clinical course, body weight and Onodera’s PNI, and treatment details of Case 1. *PNI* prognostic nutrition index

Case 1: A 66-year-old man underwent open total gastrectomy and cholecystectomy for gastric cancer, and RY reconstruction was performed. The pathological diagnosis was T4aN3aM0 Stage IIIC (Japanese classification of gastric cancer 15^th^), and he was a candidate for adjuvant chemotherapy. However, he was unable to maintain this therapy due to severe nausea. Five months later, symptoms of heartburn and nausea persisted despite the absence of chemotherapy, and esophagogastroduodenoscopy identified grade C esophagitis (Los Angeles classification). The radiographic contrast study did not show any bowel obstruction. Although he was treated with various oral medications and was hospitalized six times, central venous hyperalimentation and enteral feeding were attempted, which were ineffective. The patient had lost 19 kg of weight since the initial surgery. Finally, he underwent reoperation after confirming no recurrence at 3 years postoperatively. Intraoperative findings showed no evidence of recurrence or severe adhesions in the abdominal cavity. The gaps between the mesentery of jejunojejunostomy, Petersen’s defect, and the transverse colonic mesentery through the elevated jejunum, which were closed at the time of the initial surgery, were also closed. There were no findings of internal hernia or intestinal adhesions that could have caused intestinal obstruction. The anastomotic distance between the esophagojejunostomy and jejunojejunostomy was approximately 40 cm (Fig. 1a). After the jejunojejunostomy was dissected and closed, it was re-anastomosed to create a distance of 100 cm (Fig. 1b). The patient was discharged on the 20th postoperative day without any postoperative complications. The clinical course, body weight, albumin, Onodera’s prognostic nutrition index (PNI), and treatment details are shown in Fig. 2. Two years and 6 months after the reoperation, there was no recurrence of reflux esophagitis or related symptoms without any medications, and the patient’s weight increased from 36 to 50 kg, albumin increased from 3.1 to 4.4 mg/dL, and PNI increased from 37.8 to 53.8 (Fig. 2).

**Fig. 3:**
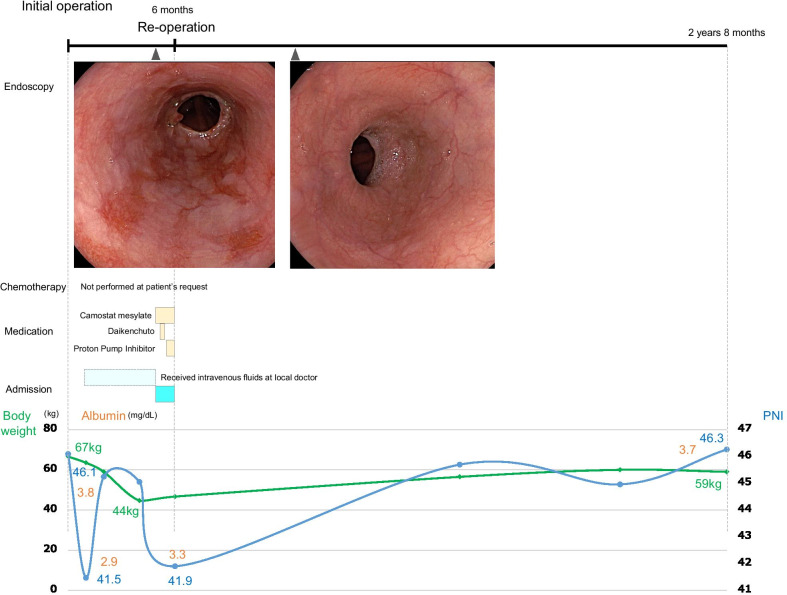
Clinical course, body weight and Onodera’s PNI, and treatment details of Case 2. *PNI* prognostic nutrition index

Case 2: A 68-year-old woman underwent open total gastrectomy and cholecystectomy for gastric cancer, and RY reconstruction was performed. She was eligible for adjuvant chemotherapy due to stage IIB (T4aN0M0) pathology, but did not wish to receive it due to the experience of severe side effects from previous postoperative chemotherapy for uterine cancer. Five months later, esophagogastroduodenoscopy identified grade C esophagitis (Los Angeles classification). The radiographic contrast study did not show any bowel obstruction. She was also refractory to medical treatment and had lost 23 kg of weight since the initial surgery. Finally, the patient was reoperated at 6 months postoperatively. Similar to Case 1, there was no recurrence or severe adhesions, and the jejunojejunostomy was re-anastomosed to increase the anastomotic distance from 40 to 100 cm. The patient was discharged on the 25th postoperative day without any postoperative complications. Two years and 8 months after the reoperation, there was no reoccurrence of reflux esophagitis without any medications, her weight increased from 44 to 59 kg, albumin increased from 3.3 to 3.7 mg/dL, and PNI increased from 41.9 to 46.3 (Fig. 3).

## Discussion

The cause of reflux esophagitis after total gastrectomy is flow of duodenal fluid, including bile and pancreatic juice [5, 6], into the esophagus. In severe cases, it causes not only heartburn and weight loss, but also Barrett’s adenocarcinoma in the long term [7]. The role of the different components of this refluxate in the pathogenesis of reflux esophagitis after total gastrectomy is only partially known. Some studies in animals have shown that trypsin, deconjugated bile salts, and lysolecithin are more damaging to the esophagus in the absence of gastric acid [8, 9]. In recent years, although there have been some reports on the use of pH and bilirubin monitors to aid in the diagnosis and understanding of pathogenesis [10–12], present cases did not receive those examinations. Although the main treatment is medication to inhibit digestive enzymes, surgery can be performed if there is resistance.

In this study, we evaluated the status of the two patients before and after reoperation from two perspectives: body weight and nutritional indicators. Body weight loss significantly influences a patient’s quality of life after gastrectomy [13]. In the present two cases, weight loss progressed due to esophagitis symptoms, with a maximum of 19 kg in Case 1 and 23 kg in Case 2, which was a decrease from the preoperative weight, but the weight increased after reoperation.

Onodera’s PNI reflects nutritional status and is calculated as follows: 10 × serum albumin (g/dL) + 0.005 × lymphocyte count (per mm^3^) [14]. Preoperative malnutrition is associated with poor prognosis after gastrectomy, and PNI has been reported to be associated with poor prognosis in the low PNI group when the cutoff was set at 44.7–47.8 [14–16]. Although PNI decreased to a minimum of 37.8 in Case 1 and 41.5 in Case 2, it increased to over 46 after reoperation, indicating that reoperation also improved nutritional status.

There were six cases of reoperation for reflux esophagitis after total gastrectomy and RY reconstruction reported from Japan and overseas (Table 1) [11, 12, 17]. In all cases, the surgery was performed safely with a relatively short operation time and a small amount of blood loss. In both our cases, although there were some adhesions in the upper abdomen, they did not affect the re-anastomosis, and the patients were discharged without any complications. The time between the initial surgery and reoperation varied between the cases. The patient in Case 1 took as long as 39 months, while the second patient was reoperated within a shorter period of 6 months. In Case 1, the pathological diagnosis was stage IIIC, indicating a high risk of recurrence, and since the possibility of passage obstruction due to recurrence could not be ruled out, the decision was made to perform reoperation after 3 years of recurrence-free confirmation. For the second case, based on the positive experience of the first, the decision to reoperate was made at a relatively early stage when the patient was resistant to medical treatment. Overall, the rapid improvement in symptoms and lack of recurrence of esophagitis after re-anastomosis suggest that transposition of jejunojejunostomy is effective, and surgical treatment may be considered at a relatively early stage for patients who are refractory to medical therapy.

**Table 1:** Cases of reoperation for reflux esophagitis after total gastrectomy and Roux-en-Y reconstruction

Reports (year)	Age/sex	Symptom	Endoscopic findings*	Time between initial surgery and reoperation (months)	Preoperative distance between esophagojejunostomy and jejunojejunostomy (cm)	Postoperative distance between esophagojejunostomy and jejunojejunostomy (cm)	Operation time (minutes)	Blood loss (ml)
Collard et al. (2002) [17]	NA	Heartburn	NA	NA	60	110	NA	NA
Collard et al. (2002) [17]	NA	Heartburn	NA	NA	60	110	NA	NA
Takahashi et al. (2008) [12]	72/M	Heartburn, body weight loss	Grade D	5	30	130	155	50
Miyamura et al. (2016) [11]	57/M	Heartburn, body weight loss	Grade D	18	30	90	115	30
Case 1	68/M	Anorexia, body weight loss	Grade C	39	40	100	110	10
Case 2	66/F	Anorexia, body weight loss	Grade C	6	40	100	163	20

*M* male, *F* female, *Los Angeles classification, NA not applicable

There are three possible causes of duodenal fluid reflux after total gastrectomy, excluding substrate occlusion mechanisms, such as recurrence or severe adhesions. The first is a reconstructive method. The Billroth II method, in which the duodenal fluid passes through the anastomosis, has been reported to cause esophagitis in 50–60% of cases, whereas the RY method has been reported to have an incidence of 10–30% [18]. Next, the shorter the anastomotic distance, the more likely reflux esophagitis occurs, and the frequency decreases when the anastomotic distance is > 40 cm [19]. However, the frequency of Roux stasis increases when the anastomotic distance exceeds 40 cm [20], and thus the appropriate anastomotic distance remains a matter of debate. Takahashi and Miyamura reported that the anastomotic distance was as short as 30 cm, and it was considered that the short distance was the cause of reflux esophagitis [11, 12]. However, our two cases revealed that reflux occurred even at a distance of 40 cm. Therefore, it is important to keep in mind that some patients may experience reflux even when the anastomotic distance is sufficient. At the time of reoperation, we set the anastomotic distance of 100 cm based on the report of Collard et al. [17], and the patients have progressed without malabsorption. According to the six reported cases, it seems that an anastomotic distance of 100 cm for re-anastomosis is a good guideline. Finally, the decrease in lower esophageal pressure due to the dissection of the lower esophagus and the negative pressure on the anastomosis due to the esophageal jejunal anastomosis being located in the thoracic cavity may also be factors in reflux [10]. In both cases, the esophageal dissection length was more than 2 cm, and the anastomosis was in the thoracic cavity, which could have contributed to reflux. Thus, in cases of total gastrectomy requiring lower esophagectomy, it may be necessary to increase the distance between the anastomoses.

## Conclusions

We present two cases of reflux esophagitis that were successfully treated by transposition of the jejunojejunal anastomosis. Transposition of the jejunojejunostomy was an effective treatment in both cases for medication-resistant severe reflux esophagitis after total gastrectomy.

## Data Availability

The data supporting the conclusions are included in the article.

## Abbreviations

PNI: Prognostic nutrition index
RY: Roux-en-Y
